# Bis[eth­yl(2-hydroxy­ethyl)aza­nium] 2,2′-disulfanediyldibenzoate

**DOI:** 10.1107/S1600536810006781

**Published:** 2010-02-27

**Authors:** Grant A. Broker, Edward R. T. Tiekink

**Affiliations:** a 5959 FM 1960 Road West, Houston, Texas 77069, USA; b Department of Chemistry, University of Malaya, 50603 Kuala Lumpur, Malaysia

## Abstract

The asymmetric unit of the title salt, 2C_4_H_12_NO^+^·C_14_H_8_O_4_S_2_^2−^, contains an eth­yl(2-hydr­oxy)aminium cation and half a 2,2′-disulfanediyldibenzoate anion, with the latter disposed about a twofold axis. The cation is a straight chain with the exception of the terminal hydr­oxy group [the N—C—C—O torsion angle is 66.5 (2)°]. A twisted conformation is found for the anion [the C—S—S—C torsion angle is 91.51 (9)° and the dihedral angle between the rings is 81.01 (4)°]. A supra­molecular chain with base vector [101] and a tubular topology is formed in the crystal structure mediated by charge-assisted O—H⋯O^−^ and N^+^—H⋯O^−^ hydrogen bonding.

## Related literature

For related studies on co-crystal/salt formation involving 2-[(2-carboxy­phen­yl)disulfan­yl]benzoic acid, see: Broker & Tiekink (2007 ▶); Broker *et al.* (2008 ▶). For software used to search the Cambridge Structural Database, see: Bruno *et al.* (2002 ▶).
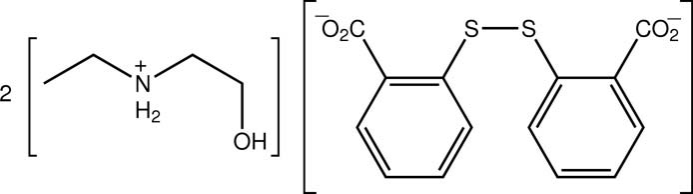


## Experimental

### 

#### Crystal data

2C_4_H_12_NO^+^·C_14_H_8_O_4_S_2_^2−^
*M* *_r_* = 484.64
Monoclinic, 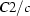

*a* = 22.949 (5) Å
*b* = 8.2429 (16) Å
*c* = 14.766 (3) Å
β = 119.80 (3)°
*V* = 2423.9 (11) Å^3^
*Z* = 4
Mo *K*α radiation
μ = 0.26 mm^−1^
*T* = 173 K
0.40 × 0.25 × 0.10 mm

#### Data collection

Rigaku AFC12/SATURN724 CCD-detector diffractometer
Absorption correction: multi-scan (*ABSCOR*; Higashi, 1995 ▶) *T*_min_ = 0.800, *T*_max_ = 1.000
7823 measured reflections
2503 independent reflections
2367 reflections with *I* > 2σ(*I*)
*R*_int_ = 0.032

#### Refinement

*R*[*F* ^2^ > 2σ(*F* ^2^)] = 0.039
*wR*(*F* ^2^) = 0.100
*S* = 1.14
2503 reflections
148 parameters
1 restraint
H-atom parameters constrained
Δρ_max_ = 0.34 e Å^−3^
Δρ_min_ = −0.22 e Å^−3^

### 

Data collection: *CrystalClear* (Rigaku/MSC, 2005 ▶); cell refinement: *CrystalClear*; data reduction: *CrystalClear*; program(s) used to solve structure: *SHELXS97* (Sheldrick, 2008 ▶); program(s) used to refine structure: *SHELXL97* (Sheldrick, 2008 ▶); molecular graphics: *ORTEPII* (Johnson, 1976 ▶) and *DIAMOND* (Brandenburg, 2006 ▶); software used to prepare material for publication: *publCIF* (Westrip, 2010 ▶).

## Supplementary Material

Crystal structure: contains datablocks global, I. DOI: 10.1107/S1600536810006781/zs2030sup1.cif

Structure factors: contains datablocks I. DOI: 10.1107/S1600536810006781/zs2030Isup2.hkl

Additional supplementary materials:  crystallographic information; 3D view; checkCIF report

## Figures and Tables

**Table 1:** Hydrogen-bond geometry (Å, °)

*D*—H⋯*A*	*D*—H	H⋯*A*	*D*⋯*A*	*D*—H⋯*A*
N1—H1*A*⋯O1^i^	0.92	1.94	2.840 (2)	164
N1—H1*B*⋯O2	0.92	1.85	2.7617 (19)	171
O3—H3⋯O2^i^	0.84	1.92	2.763 (2)	177

Symmetry code: (i) 
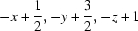
.

**Fig. 1.**
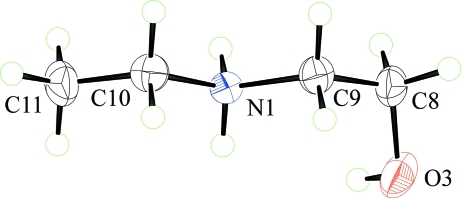
Molecular structure of the cation in (I) showing atom-labelling scheme and displacement ellipsoids at the 50% probability level.

**Fig. 2.**
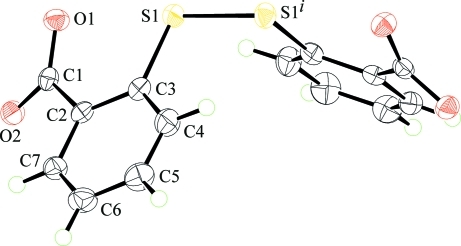
Molecular structure of the anion in (I) showing atom-labelling scheme and displacement ellipsoids at the 50% probability level. The anion has crystallographic 2-fold symmetry; i: -x, y, 1/2-z.

**Fig. 3.**
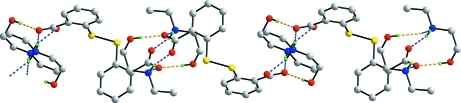
Supramolecular chain formation in (I) mediated by charge-assisted O–H···O- (orange dashed lines) and N+–H···O- (blue dashed lines) hydrogen bonding. Colour code: S, yellow; O, red; N, blue; C, grey; H, green.

**Fig. 4.**
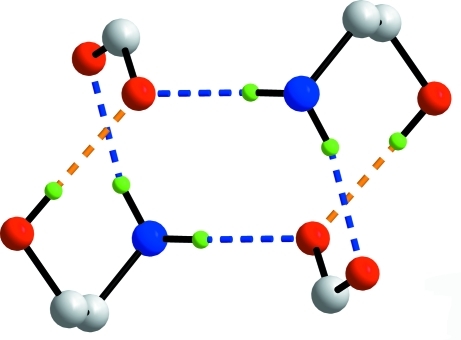
An end-on view of the supramolecular chain shown in Fig. 3 highlighting the tubular topology. The charge-assisted O–H···O- and N+–H···O- hydrogen-bonding interactions are indicated by orange and blue dashed lines, respectively. Colour code: S, yellow; O, red; N, blue; C, grey; H, green.

## supplementary crystallographic information

### Comment

The title salt, (I), was obtained during crystallisation experiments involving 2-[(2-carboxyphenyl)disulfanyl]benzoic acid with various N-containing species (Broker & Tiekink, 2007; Broker *et al.*, 2008). The asymmetric unit comprises an aminium cation (Fig. 1) and half a dithiodibenzoate anion (Fig. 2), with the latter disposed about a crystallographic 2-fold axis. The cation is linear with the exception of the terminal hydroxy group which is twisted out of the chain as seen in the O3–C8–C9–N1 torsion angle [66.5 (2)°]. Confirmation of protonation of the amine-N1 atom during crystallisation is seen in the pattern of hydrogen-bonding interactions (see below). A search of the CSD (Bruno *et al.*, 2002) suggests that this is the first structural characterisation reported for the ethyl(2-hydroxyethyl)aminium cation. The dithiodibenzoate anion is twisted [torsion angle C3–S1–S1*^i^*–C3*^i^* = 91.51 (9)°: for symmetry code *i*, -*x*, *y*, 1/2-*z*] in accord with expectation, with the conformation stabilised by an intramolecular S···O interaction of 2.7351 (16) Å (Broker & Tiekink (2007). The carboxylate group is twisted out of the plane of the benzene ring to which it is connected with the C3–C2–C1–O1 torsion angle being -25.0 (2) °. Confirmation of deprotonation of the carboxylic acid is consistent with the observed near equivalence of the C1–O1 and C1–O2 bond distances [1.2499 (19) and 1.270 (2) Å] with the weaker C1–O2 bond correlated to the participation of the O2 atom in two hydrogen bonding interactions compared to one for the O1 atom. The crystal packing is dominated by charge-assisted O–H···O^-^ and N^+^–H···O^-^ hydrogen bonding (Table 1). Each of the aminium-H atoms connects to a carboxylate-O atom and the O2 atom is also hydrogen-bonded to the hydroxy group. The result of these interactions is a supramolecular chain with base vector [1 0 1] (Fig. 3), which has a tubular topology (Fig. 4).

### Experimental

The title salt (I) was obtained by dissolving 2-[(2-carboxyphenyl)disulfanyl]benzoic acid (0.100 g, Fluka) in ethanol (20 ml) to which was added the amine in 1:1, 1:2 and 1:3 stoichiometric ratios in three separate experiments. Regardless of the stoichiometry, only crystals of (I) were harvested as proved by multiple unit cell determinations, m.p. 429–431 K

### Refinement

The H-atoms were located from difference maps but placed in their idealised positions (O–H = 0.84 Å, N–H = 0.92 Å, and C–H 0.95-0.99 Å) and were included in the refinement in the riding model approximation with *U*_iso_(H) set to 1.2-1.5*U*_eq_(carrier atom).

### Crystal data

**Table:**

2C_4_H_12_NO^+^·C_14_H_8_O_4_S_2_^2^^−^	*F*(000) = 1032
*M**_r_* = 484.64	*D*_x_ = 1.328 Mg m^−^^3^
Monoclinic, *C*2/*c*	Mo *K*α radiation, λ = 0.71073 Å
Hall symbol: -C 2yc	Cell parameters from 5747 reflections
*a* = 22.949 (5) Å	θ = 3.6–30.5°
*b* = 8.2429 (16) Å	µ = 0.26 mm^−^^1^
*c* = 14.766 (3) Å	*T* = 173 K
β = 119.80 (3)°	Block, colourless
*V* = 2423.9 (11) Å^3^	0.40 × 0.25 × 0.10 mm
*Z* = 4	

### Data collection

**Table:**

Rigaku AFC12K/SATURN724 CCD-detector diffractometer	2503 independent reflections
Radiation source: fine-focus sealed tube	2367 reflections with *I* > 2σ(*I*)
graphite	*R*_int_ = 0.032
ω scans	θ_max_ = 26.5°, θ_min_ = 3.6°
Absorption correction: multi-scan (*ABSCOR*; Higashi, 1995)	*h* = −28→25
*T*_min_ = 0.800, *T*_max_ = 1.000	*k* = −10→10
7823 measured reflections	*l* = −18→18

### Refinement

**Table:**

Refinement on *F*^2^	Primary atom site location: structure-invariant direct methods
Least-squares matrix: full	Secondary atom site location: difference Fourier map
*R*[*F*^2^ > 2σ(*F*^2^)] = 0.039	Hydrogen site location: inferred from neighbouring sites
*wR*(*F*^2^) = 0.100	H-atom parameters constrained
*S* = 1.14	*w* = 1/[σ^2^(*F*_o_^2^) + (0.0437*P*)^2^ + 1.6814*P*] where *P* = (*F*_o_^2^ + 2*F*_c_^2^)/3
2503 reflections	(Δ/σ)_max_ = 0.001
148 parameters	Δρ_max_ = 0.34 e Å^−^^3^
1 restraint	Δρ_min_ = −0.22 e Å^−^^3^

### Special details

**Table:**

Geometry. All esds (except the esd in the dihedral angle between two l.s. planes) are estimated using the full covariance matrix. The cell esds are taken into account individually in the estimation of esds in distances, angles and torsion angles; correlations between esds in cell parameters are only used when they are defined by crystal symmetry. An approximate (isotropic) treatment of cell esds is used for estimating esds involving l.s. planes.
Refinement. Refinement of *F*^2^ against ALL reflections. The weighted *R*-factor wR and goodness of fit *S* are based on *F*^2^, conventional *R*-factors *R* are based on *F*, with *F* set to zero for negative *F*^2^. The threshold expression of *F*^2^ > σ(*F*^2^) is used only for calculating *R*-factors(gt) etc. and is not relevant to the choice of reflections for refinement. *R*-factors based on *F*^2^ are statistically about twice as large as those based on *F*, and *R*- factors based on ALL data will be even larger.

### Fractional atomic coordinates and isotropic or equivalent isotropic displacement parameters (Å^2^)

**Table:**

	*x*	*y*	*z*	*U*_iso_*/*U*_eq_	
S1	0.04587 (2)	0.42885 (5)	0.31713 (3)	0.02621 (14)	
O1	0.17238 (6)	0.44668 (13)	0.48609 (9)	0.0272 (3)	
O2	0.21265 (6)	0.67861 (14)	0.57133 (9)	0.0269 (3)	
O3	0.17235 (7)	0.9908 (2)	0.29494 (10)	0.0453 (4)	
H3	0.2072	0.9376	0.3338	0.068*	
N1	0.20710 (7)	1.00243 (16)	0.51963 (10)	0.0231 (3)	
H1A	0.2410	1.0242	0.5052	0.028*	
H1B	0.2069	0.8923	0.5296	0.028*	
C1	0.16465 (8)	0.58217 (18)	0.51667 (12)	0.0223 (3)	
C2	0.09497 (8)	0.63551 (18)	0.48785 (12)	0.0222 (3)	
C3	0.03728 (8)	0.57605 (18)	0.39935 (12)	0.0236 (3)	
C4	−0.02554 (9)	0.6307 (2)	0.37906 (14)	0.0329 (4)	
H4	−0.0647	0.5931	0.3187	0.039*	
C5	−0.03181 (10)	0.7382 (2)	0.44505 (15)	0.0376 (4)	
H5	−0.0752	0.7728	0.4303	0.045*	
C6	0.02468 (9)	0.7965 (2)	0.53293 (14)	0.0335 (4)	
H6	0.0203	0.8701	0.5787	0.040*	
C7	0.08727 (9)	0.74627 (19)	0.55301 (13)	0.0269 (4)	
H7	0.1261	0.7877	0.6124	0.032*	
C8	0.12733 (9)	0.9551 (2)	0.33125 (14)	0.0351 (4)	
H8A	0.1298	0.8376	0.3468	0.042*	
H8B	0.0810	0.9794	0.2753	0.042*	
C9	0.14185 (9)	1.0497 (2)	0.42769 (13)	0.0306 (4)	
H9A	0.1427	1.1670	0.4139	0.037*	
H9B	0.1053	1.0309	0.4437	0.037*	
C10	0.22223 (9)	1.0870 (2)	0.61827 (14)	0.0316 (4)	
H10A	0.1855	1.0671	0.6336	0.038*	
H10B	0.2252	1.2053	0.6099	0.038*	
C11	0.28740 (10)	1.0267 (3)	0.70735 (15)	0.0430 (5)	
H11A	0.2967	1.0832	0.7716	0.065*	
H11B	0.3238	1.0477	0.6924	0.065*	
H11C	0.2842	0.9098	0.7162	0.065*	

### Atomic displacement parameters (Å^2^)

**Table:**

	*U*^11^	*U*^22^	*U*^33^	*U*^12^	*U*^13^	*U*^23^
S1	0.0231 (2)	0.0274 (2)	0.0238 (2)	0.00348 (15)	0.00832 (17)	−0.00108 (15)
O1	0.0244 (6)	0.0241 (6)	0.0319 (6)	0.0018 (5)	0.0131 (5)	−0.0030 (5)
O2	0.0229 (6)	0.0252 (6)	0.0285 (6)	−0.0020 (5)	0.0097 (5)	−0.0005 (5)
O3	0.0373 (8)	0.0698 (10)	0.0298 (7)	0.0153 (7)	0.0174 (6)	0.0157 (7)
N1	0.0237 (7)	0.0218 (7)	0.0246 (7)	0.0019 (5)	0.0126 (6)	0.0006 (5)
C1	0.0239 (8)	0.0234 (8)	0.0194 (7)	0.0006 (6)	0.0107 (7)	0.0047 (6)
C2	0.0239 (8)	0.0194 (7)	0.0243 (8)	0.0022 (6)	0.0128 (7)	0.0051 (6)
C3	0.0234 (8)	0.0231 (8)	0.0243 (8)	0.0029 (6)	0.0118 (7)	0.0039 (6)
C4	0.0237 (9)	0.0377 (10)	0.0326 (9)	0.0040 (8)	0.0105 (8)	−0.0010 (8)
C5	0.0288 (10)	0.0427 (10)	0.0431 (10)	0.0101 (8)	0.0192 (9)	−0.0002 (9)
C6	0.0386 (10)	0.0316 (9)	0.0370 (9)	0.0056 (8)	0.0239 (8)	−0.0013 (8)
C7	0.0307 (9)	0.0239 (8)	0.0261 (8)	−0.0002 (7)	0.0142 (7)	0.0004 (7)
C8	0.0273 (9)	0.0471 (10)	0.0247 (9)	0.0033 (8)	0.0083 (7)	0.0042 (8)
C9	0.0256 (9)	0.0318 (9)	0.0311 (9)	0.0069 (7)	0.0117 (8)	0.0062 (7)
C10	0.0373 (10)	0.0296 (9)	0.0323 (9)	−0.0014 (7)	0.0207 (8)	−0.0073 (7)
C11	0.0356 (11)	0.0634 (13)	0.0266 (9)	−0.0058 (10)	0.0128 (8)	−0.0115 (9)

### Geometric parameters (Å, °)

**Table:**

S1—C3	1.7953 (16)	C5—C6	1.387 (3)
S1—S1^i^	2.0528 (13)	C5—H5	0.9500
O1—C1	1.2499 (19)	C6—C7	1.378 (2)
O2—C1	1.270 (2)	C6—H6	0.9500
O3—C8	1.411 (2)	C7—H7	0.9500
O3—H3	0.8401	C8—C9	1.509 (3)
N1—C9	1.489 (2)	C8—H8A	0.9900
N1—C10	1.492 (2)	C8—H8B	0.9900
N1—H1A	0.9200	C9—H9A	0.9900
N1—H1B	0.9200	C9—H9B	0.9900
C1—C2	1.502 (2)	C10—C11	1.503 (3)
C2—C7	1.400 (2)	C10—H10A	0.9900
C2—C3	1.407 (2)	C10—H10B	0.9900
C3—C4	1.393 (2)	C11—H11A	0.9800
C4—C5	1.377 (3)	C11—H11B	0.9800
C4—H4	0.9500	C11—H11C	0.9800
			
C3—S1—S1^i^	104.39 (6)	C6—C7—H7	119.3
C8—O3—H3	105.1	C2—C7—H7	119.3
C9—N1—C10	114.12 (13)	O3—C8—C9	113.00 (16)
C9—N1—H1A	108.7	O3—C8—H8A	109.0
C10—N1—H1A	108.7	C9—C8—H8A	109.0
C9—N1—H1B	108.7	O3—C8—H8B	109.0
C10—N1—H1B	108.7	C9—C8—H8B	109.0
H1A—N1—H1B	107.6	H8A—C8—H8B	107.8
O1—C1—O2	123.69 (15)	N1—C9—C8	112.07 (14)
O1—C1—C2	118.79 (14)	N1—C9—H9A	109.2
O2—C1—C2	117.52 (14)	C8—C9—H9A	109.2
C7—C2—C3	118.91 (15)	N1—C9—H9B	109.2
C7—C2—C1	118.46 (14)	C8—C9—H9B	109.2
C3—C2—C1	122.62 (14)	H9A—C9—H9B	107.9
C4—C3—C2	118.84 (15)	N1—C10—C11	110.34 (14)
C4—C3—S1	121.51 (13)	N1—C10—H10A	109.6
C2—C3—S1	119.63 (12)	C11—C10—H10A	109.6
C5—C4—C3	121.12 (17)	N1—C10—H10B	109.6
C5—C4—H4	119.4	C11—C10—H10B	109.6
C3—C4—H4	119.4	H10A—C10—H10B	108.1
C4—C5—C6	120.48 (17)	C10—C11—H11A	109.5
C4—C5—H5	119.8	C10—C11—H11B	109.5
C6—C5—H5	119.8	H11A—C11—H11B	109.5
C7—C6—C5	119.15 (16)	C10—C11—H11C	109.5
C7—C6—H6	120.4	H11A—C11—H11C	109.5
C5—C6—H6	120.4	H11B—C11—H11C	109.5
C6—C7—C2	121.48 (16)		
			
O1—C1—C2—C7	153.69 (15)	C2—C3—C4—C5	−1.4 (3)
O2—C1—C2—C7	−26.3 (2)	S1—C3—C4—C5	177.14 (15)
O1—C1—C2—C3	−25.0 (2)	C3—C4—C5—C6	0.9 (3)
O2—C1—C2—C3	155.09 (14)	C4—C5—C6—C7	0.5 (3)
C7—C2—C3—C4	0.6 (2)	C5—C6—C7—C2	−1.3 (3)
C1—C2—C3—C4	179.26 (15)	C3—C2—C7—C6	0.7 (2)
C7—C2—C3—S1	−177.96 (12)	C1—C2—C7—C6	−177.99 (15)
C1—C2—C3—S1	0.7 (2)	C10—N1—C9—C8	−177.21 (14)
S1^i^—S1—C3—C4	16.34 (15)	O3—C8—C9—N1	−66.5 (2)
S1^i^—S1—C3—C2	−165.14 (11)	C9—N1—C10—C11	177.61 (15)

Symmetry codes: (i) −*x*, *y*, −*z*+1/2.

### Hydrogen-bond geometry (Å, °)

**Table:**

*D*—H···*A*	*D*—H	H···*A*	*D*···*A*	*D*—H···*A*
N1—H1A···O1^ii^	0.92	1.94	2.840 (2)	164
N1—H1B···O2	0.92	1.85	2.7617 (19)	171
O3—H3···O2^ii^	0.84	1.92	2.763 (2)	177

Symmetry codes: (ii) −*x*+1/2, −*y*+3/2, −*z*+1.
